# Analysis of survival data from trials with non-proportional hazards: an empirical comparison of methods

**DOI:** 10.1186/1745-6215-14-S1-O38

**Published:** 2013-11-29

**Authors:** Patrick Royston, Yinghui Wei, Jayne Tierney, Mahesh Parmar

**Affiliations:** 1 MRC Clinical Trials Unit Hub for Trials Methodology Research, London, UK

## Introduction

Non-proportional hazards are increasingly observed in publications of large trials. However, to use or not to use a method accounting for non-proportional hazards may be a difficult decision, in the absence of appraisals of existing methods and the uncertainty in whether results provide different or more insights into the clinical questions.

## Methods

We analyse survival data reconstructed from two trials publications which reported extreme non-proportional hazards (with respective p-values as 9.19E-13 and 1.344E-24 in non-PH Grambsch-Thernau test). We use the Cox proportional hazards model, flexible parametric model and accelerated failure time model to estimate the time-independent hazard ratio, between-arm difference in restricted mean survival time (RMST) and acceleration factor as respective summary treatment effect measures. Estimation of the latter two measures does not require PH. We also analyse the hazard ratio and difference in RMST as time-dependent effects.

## Results

In this empirical study, the three summary measures produce broadly similar results on the evidence against the null hypothesis. Both the Cox model and the flexible model are able to incorporate time-dependent treatment effects to account for non-proportional hazards. Flexible parametric models support plotting of the time-dependent hazard ratio and time-dependent difference in RMST, which provides useful insights into how the treatment effect measure may change with time.

## Conclusions

Although p-values for the three summary measures have similar influence on the statistical significance of treatment effects, using the difference in RMST as the summary measure emphasizes the often-neglected time-horizon and allows more intuitive interpretation of the magnitude of treatment effects.

